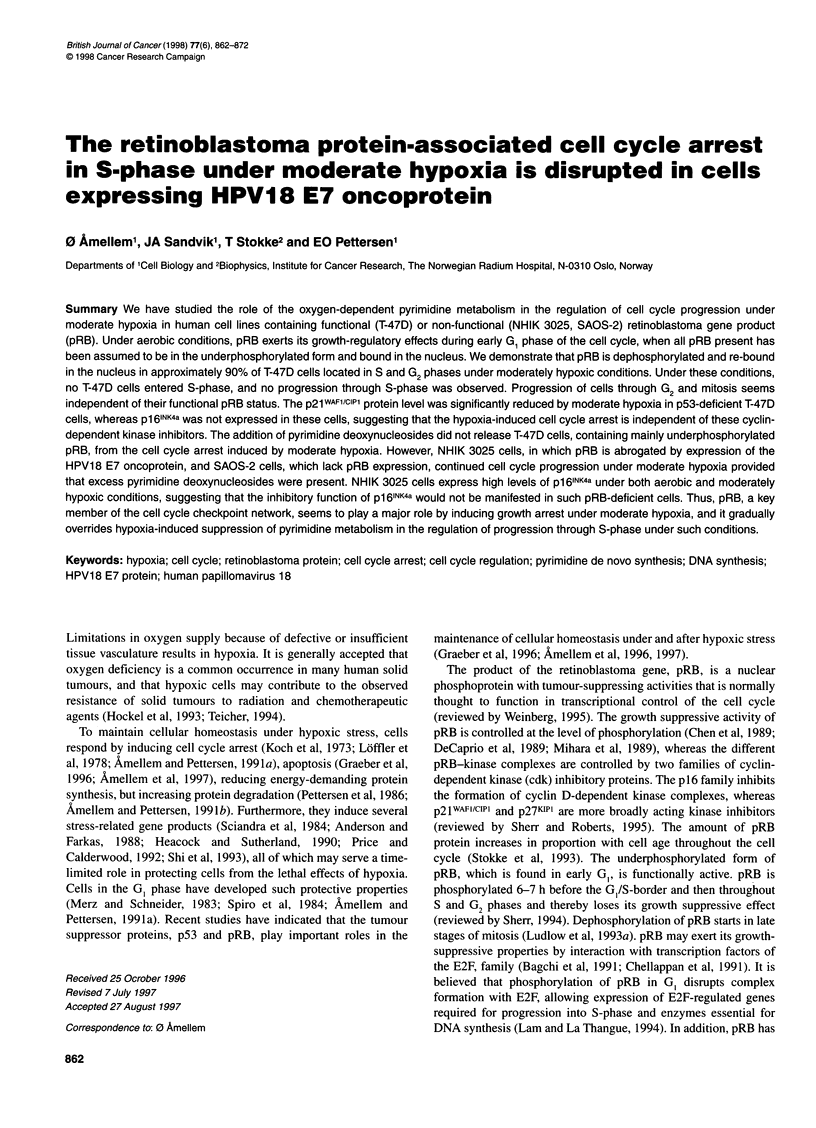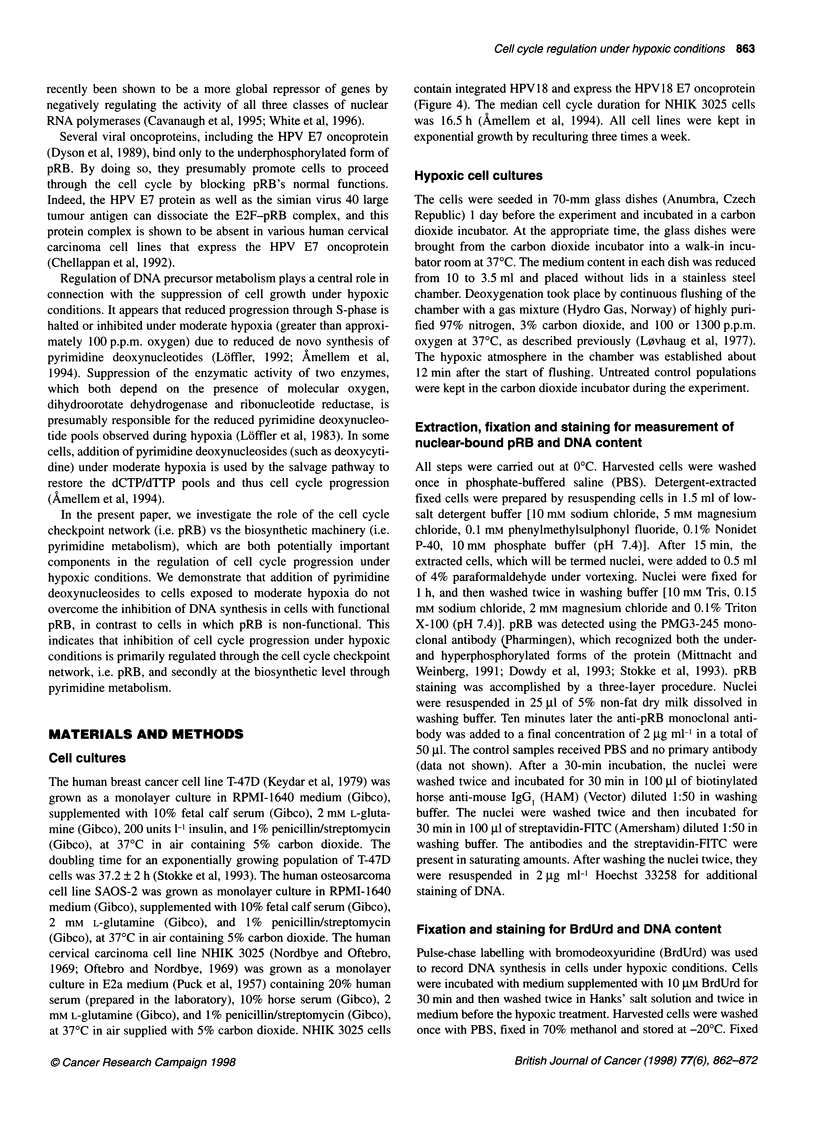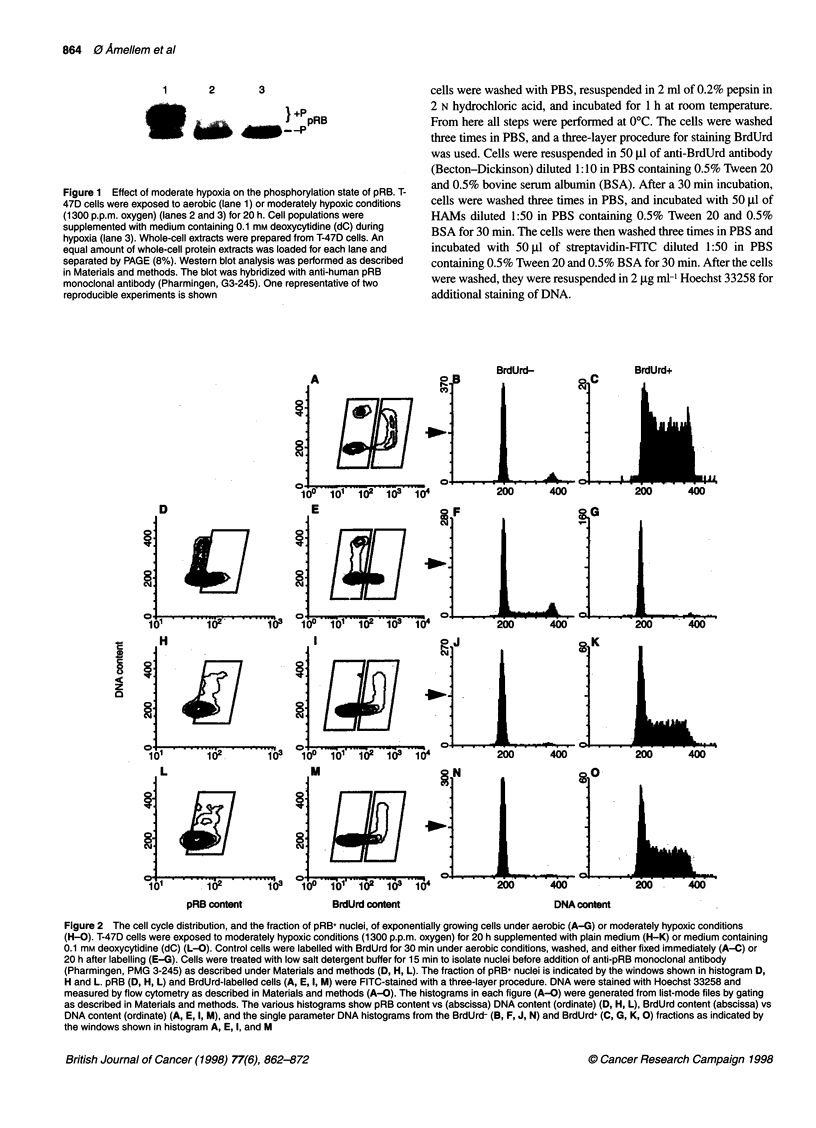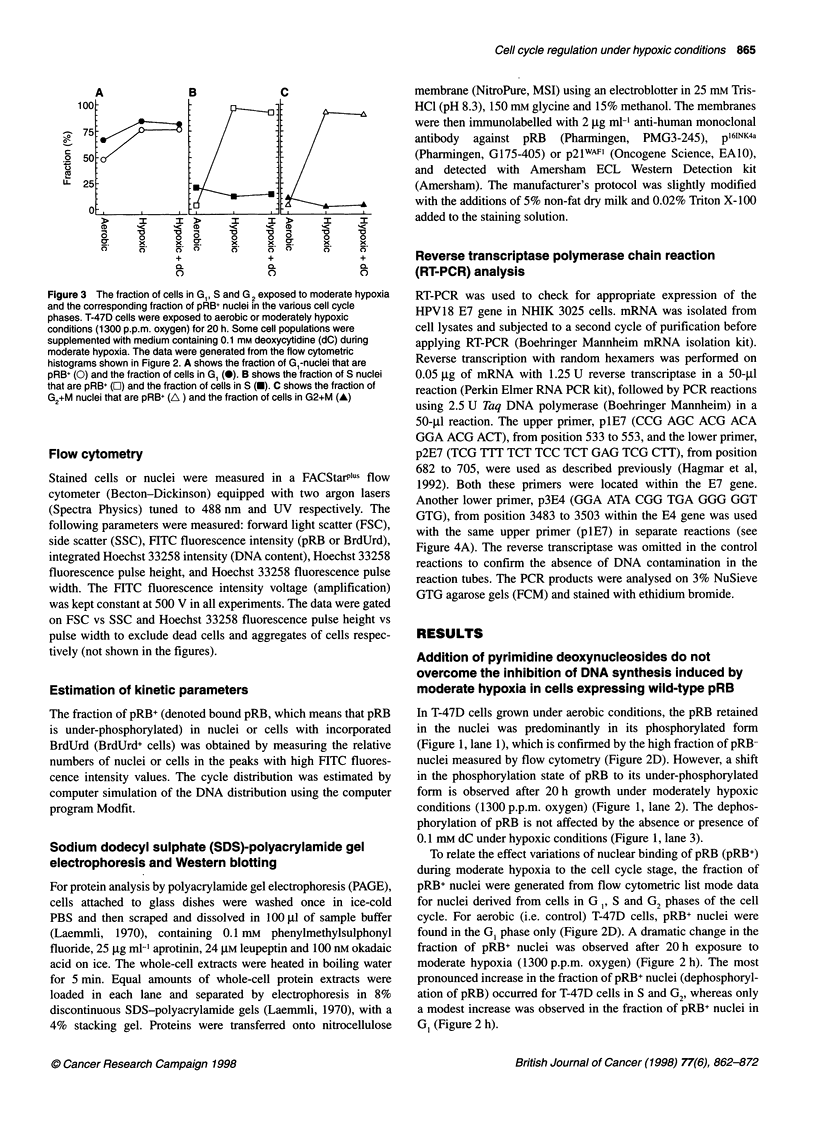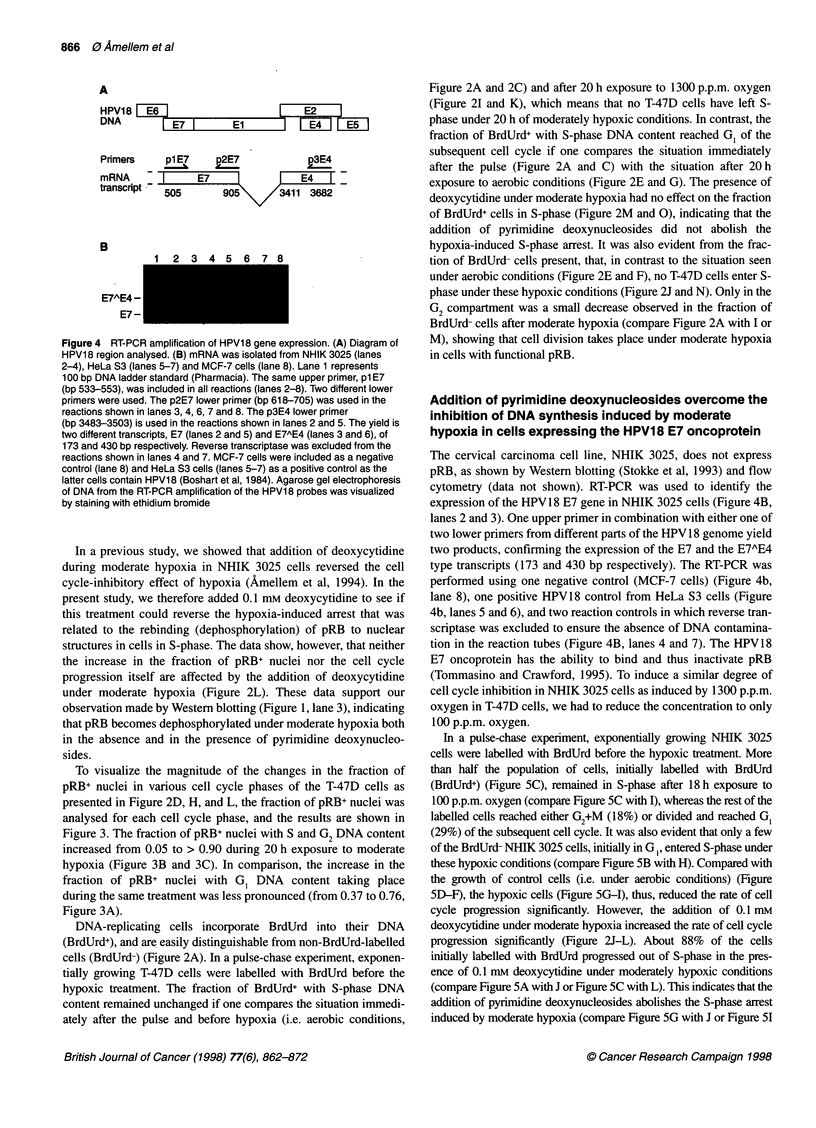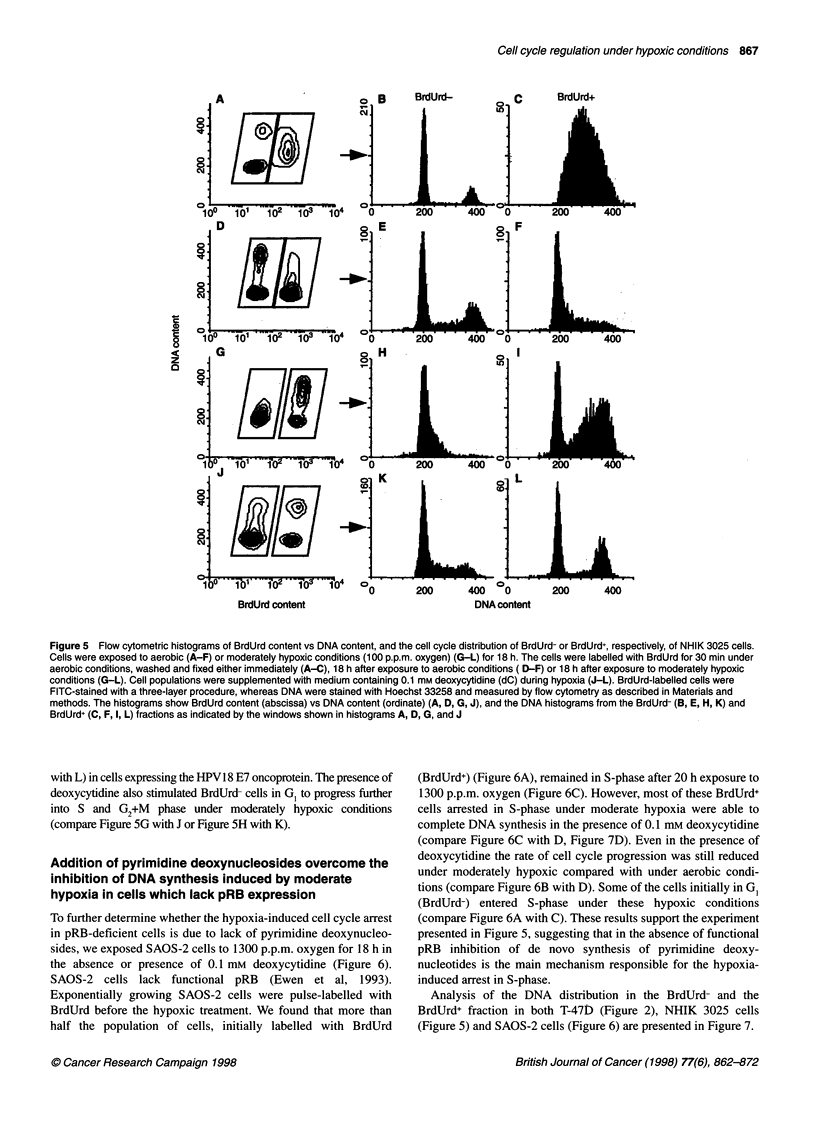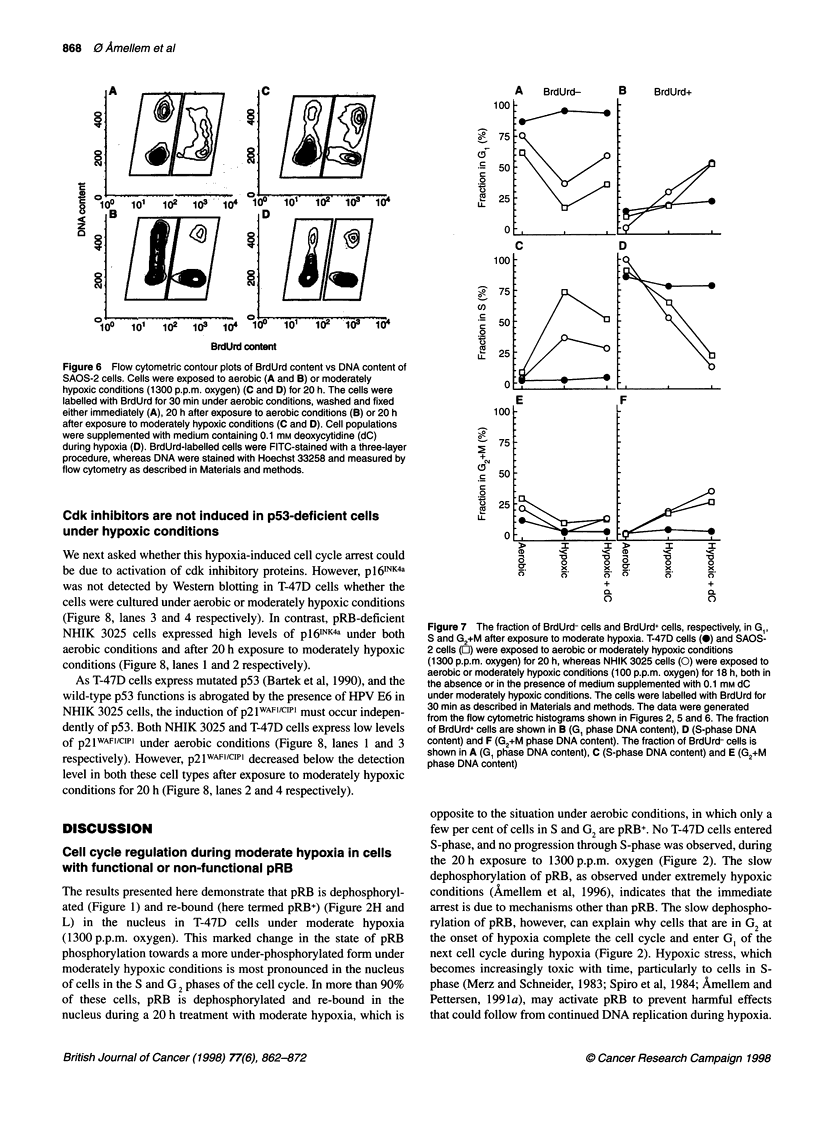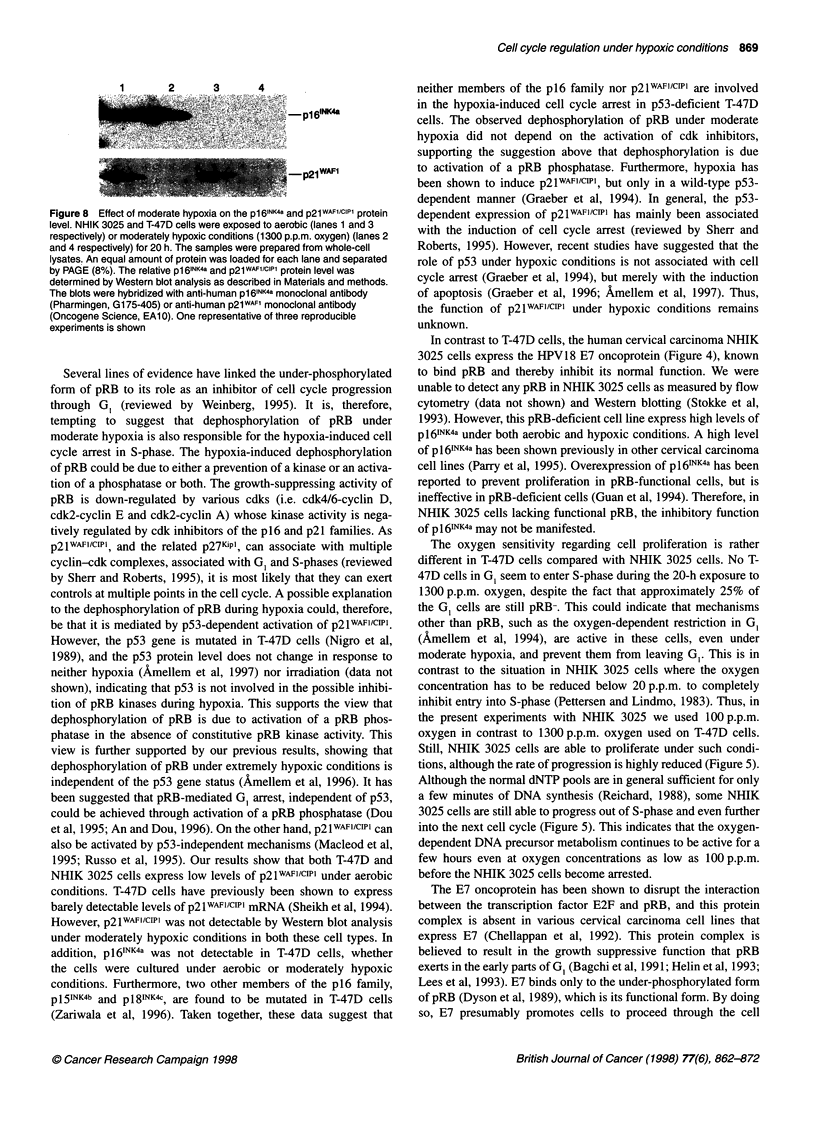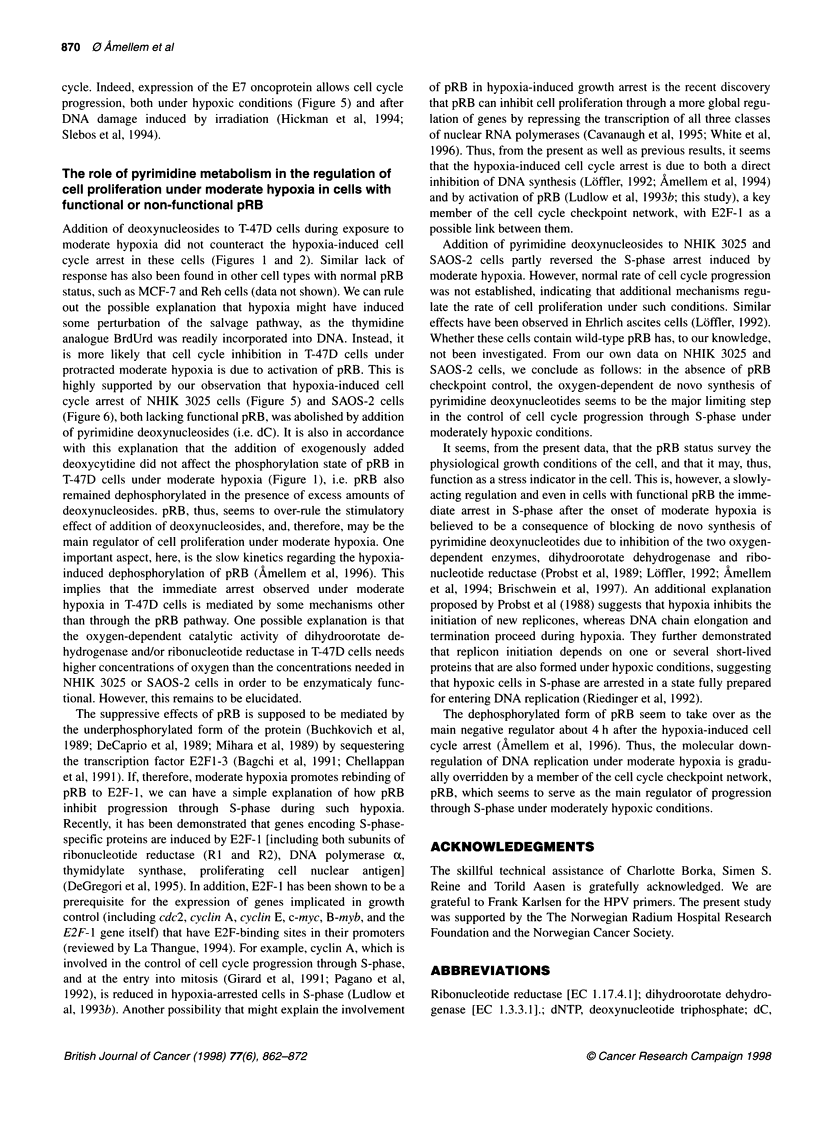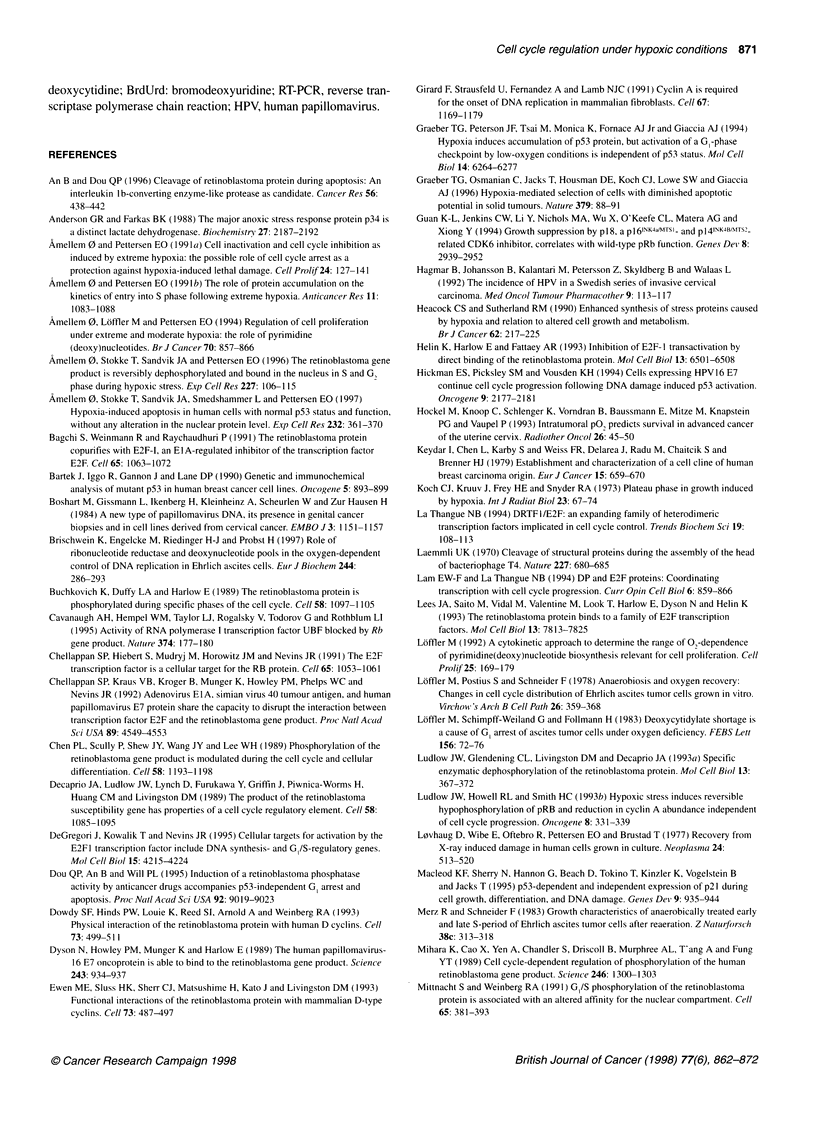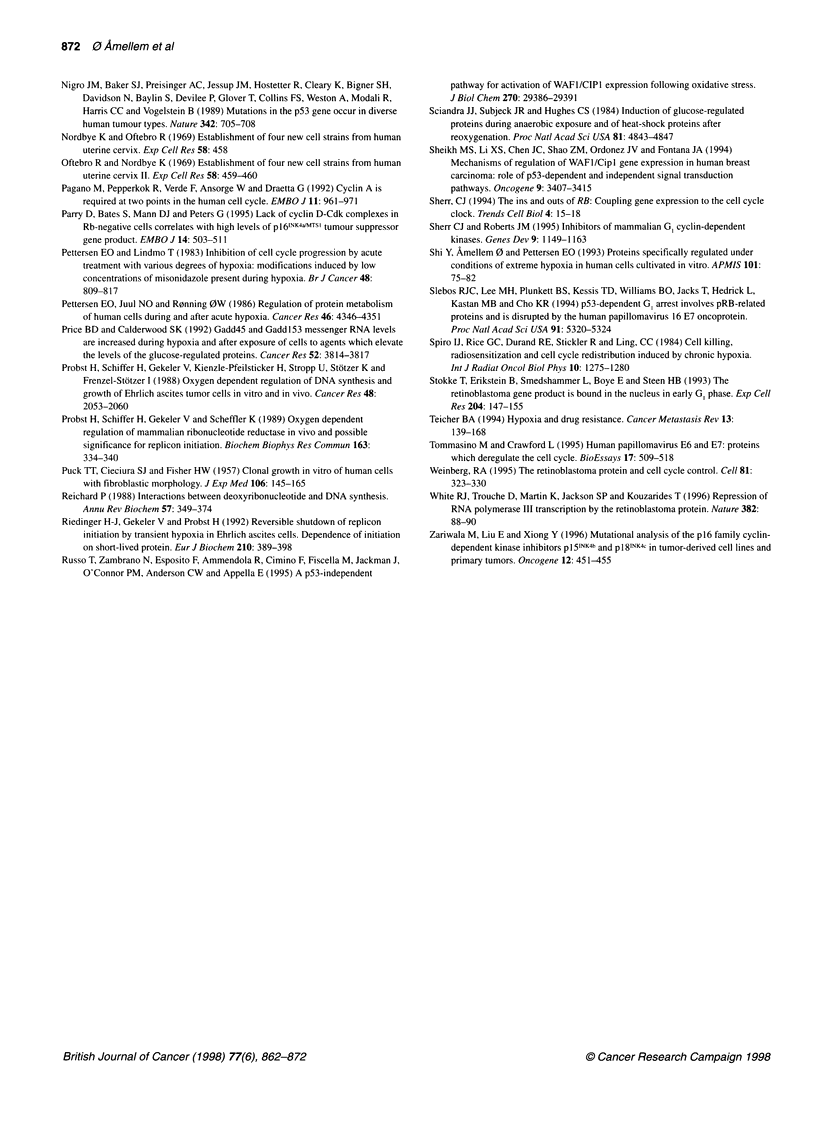# The retinoblastoma protein-associated cell cycle arrest in S-phase under moderate hypoxia is disrupted in cells expressing HPV18 E7 oncoprotein.

**DOI:** 10.1038/bjc.1998.143

**Published:** 1998-03

**Authors:** O. Amellem, J. A. Sandvik, T. Stokke, E. O. Pettersen

**Affiliations:** Department of Cell Biology, Institute for Cancer Research, The Norwegian Radium Hospital, Oslo.

## Abstract

**Images:**


British Journal of Cancer (1998) 77(6), 862–872
© 1998 Cancer Research Campaign

### The retinoblastoma protein-associated cell cycle arrest in S-phase under moderate hypoxia is disrupted in cells expressing HPV18 E7 oncoprotein

Ø Åmellem[1], JA Sandvik[1], T Stokke[2] and EO Pettersen[1]

Departments of [1]Cell Biology and [2]Biophysics, Institute for Cancer Research, The Norwegian Radium Hospital, N-0310 Oslo, Norway

**Summary** We have studied the role of the oxygen-dependent pyrimidine metabolism in the regulation of cell cycle progression under moderate hypoxia in human cell lines containing functional (T-47D) or non-functional (NHIK 3025, SAOS-2) retinoblastoma gene product (pRB). Under aerobic conditions, pRB exerts its growth-regulatory effects during early $G_1$ phase of the cell cycle, when all pRB present has been assumed to be in the underphosphorylated form and bound in the nucleus. We demonstrate that pRB is dephosphorylated and re-bound in the nucleus in approximately 90% of T-47D cells located in S and $G_2$ phases under moderately hypoxic conditions. Under these conditions, no T-47D cells entered S-phase, and no progression through S-phase was observed. Progression of cells through $G_2$ and mitosis seems independent of their functional pRB status. The p21$^{WAF1/CIP1}$ protein level was significantly reduced by moderate hypoxia in p53-deficient T-47D cells, whereas p16$^{INK4a}$ was not expressed in these cells, suggesting that the hypoxia-induced cell cycle arrest is independent of these cyclin-dependent kinase inhibitors. The addition of pyrimidine deoxynucleosides did not release T-47D cells, containing mainly underphosphorylated pRB, from the cell cycle arrest induced by moderate hypoxia. However, NHIK 3025 cells, in which pRB is abrogated by expression of the HPV18 E7 oncoprotein, and SAOS-2 cells, which lack pRB expression, continued cell cycle progression under moderate hypoxia provided that excess pyrimidine deoxynucleosides were present. NHIK 3025 cells express high levels of p16$^{INK4a}$ under both aerobic and moderately hypoxic conditions, suggesting that the inhibitory function of p16$^{INK4a}$ would not be manifested in such pRB-deficient cells. Thus, pRB, a key member of the cell cycle checkpoint network, seems to play a major role by inducing growth arrest under moderate hypoxia, and it gradually overrides hypoxia-induced suppression of pyrimidine metabolism in the regulation of progression through S-phase under such conditions.

**Keywords:** hypoxia; cell cycle; retinoblastoma protein; cell cycle arrest; cell cycle regulation; pyrimidine de novo synthesis; DNA synthesis; HPV18 E7 protein; human papillomavirus 18

Limitations in oxygen supply because of defective or insufficient tissue vasculature results in hypoxia. It is generally accepted that oxygen deficiency is a common occurrence in many human solid tumours, and that hypoxic cells may contribute to the observed resistance of solid tumours to radiation and chemotherapeutic agents (Hockel et al, 1993; Teicher, 1994).

To maintain cellular homeostasis under hypoxic stress, cells respond by inducing cell cycle arrest (Koch et al, 1973; Löffler et al, 1978; Åmellem and Pettersen, 1991*a*), apoptosis (Graeber et al, 1996; Åmellem et al, 1997), reducing energy-demanding protein synthesis, but increasing protein degradation (Pettersen et al, 1986; Åmellem and Pettersen, 1991*b*). Furthermore, they induce several stress-related gene products (Sciandra et al, 1984; Anderson and Farkas, 1988; Heacock and Sutherland, 1990; Price and Calderwood, 1992; Shi et al, 1993), all of which may serve a time-limited role in protecting cells from the lethal effects of hypoxia. Cells in the $G_1$ phase have developed such protective properties (Merz and Schneider, 1983; Spiro et al, 1984; Åmellem and Pettersen, 1991a). Recent studies have indicated that the tumour suppressor proteins, p53 and pRB, play important roles in the maintenance of cellular homeostasis under and after hypoxic stress (Graeber et al, 1996; Åmellem et al, 1996, 1997).

The product of the retinoblastoma gene, pRB, is a nuclear phosphoprotein with tumour-suppressing activities that is normally thought to function in transcriptional control of the cell cycle (reviewed by Weinberg, 1995). The growth suppressive activity of pRB is controlled at the level of phosphorylation (Chen et al, 1989; DeCaprio et al, 1989; Mihara et al, 1989), whereas the different pRB–kinase complexes are controlled by two families of cyclin-dependent kinase (cdk) inhibitory proteins. The p16 family inhibits the formation of cyclin D-dependent kinase complexes, whereas p21$^{WAF1/CIP1}$ and p27$^{KIP1}$ are more broadly acting kinase inhibitors (reviewed by Sherr and Roberts, 1995). The amount of pRB protein increases in proportion with cell age throughout the cell cycle (Stokke et al, 1993). The underphosphorylated form of pRB, which is found in early $G_1$, is functionally active. pRB is phosphorylated 6–7 h before the $G_1$/S-border and then throughout S and $G_2$ phases and thereby loses its growth suppressive effect (reviewed by Sherr, 1994). Dephosphorylation of pRB starts in late stages of mitosis (Ludlow et al, 1993*a*). pRB may exert its growth-suppressive properties by interaction with transcription factors of the E2F, family (Bagchi et al, 1991; Chellappan et al, 1991). It is believed that phosphorylation of pRB in $G_1$ disrupts complex formation with E2F, allowing expression of E2F-regulated genes required for progression into S-phase and enzymes essential for DNA synthesis (Lam and La Thangue, 1994). In addition, pRB has

*Received 25 Ocrober 1996*
*Revised 7 July 1997*
*Accepted 27 August 1997*

*Correspondence to*: Ø Åmellem recently been shown to be a more global repressor of genes by negatively regulating the activity of all three classes of nuclear RNA polymerases (Cavanaugh et al, 1995; White et al, 1996).

Several viral oncoproteins, including the HPV E7 oncoprotein (Dyson et al, 1989), bind only to the underphosphorylated form of pRB. By doing so, they presumably promote cells to proceed through the cell cycle by blocking pRB's normal functions. Indeed, the HPV E7 protein as well as the simian virus 40 large tumour antigen can dissociate the E2F–pRB complex, and this protein complex is shown to be absent in various human cervical carcinoma cell lines that express the HPV E7 oncoprotein (Chellappan et al, 1992).

Regulation of DNA precursor metabolism plays a central role in connection with the suppression of cell growth under hypoxic conditions. It appears that reduced progression through S-phase is halted or inhibited under moderate hypoxia (greater than approximately 100 p.p.m. oxygen) due to reduced de novo synthesis of pyrimidine deoxynucleotides (Löffler, 1992; Åmellem et al, 1994). Suppression of the enzymatic activity of two enzymes, which both depend on the presence of molecular oxygen, dihydroorotate dehydrogenase and ribonucleotide reductase, is presumably responsible for the reduced pyrimidine deoxynucleotide pools observed during hypoxia (Löffler et al, 1983). In some cells, addition of pyrimidine deoxynucleosides (such as deoxycytidine) under moderate hypoxia is used by the salvage pathway to restore the dCTP/dTTP pools and thus cell cycle progression (Åmellem et al, 1994).

In the present paper, we investigate the role of the cell cycle checkpoint network (i.e. pRB) vs the biosynthetic machinery (i.e. pyrimidine metabolism), which are both potentially important components in the regulation of cell cycle progression under hypoxic conditions. We demonstrate that addition of pyrimidine deoxynucleosides to cells exposed to moderate hypoxia do not overcome the inhibition of DNA synthesis in cells with functional pRB, in contrast to cells in which pRB is non-functional. This indicates that inhibition of cell cycle progression under hypoxic conditions is primarily regulated through the cell cycle checkpoint network, i.e. pRB, and secondly at the biosynthetic level through pyrimidine metabolism.

### MATERIALS AND METHODS

### Cell cultures

The human breast cancer cell line T-47D (Keydar et al, 1979) was grown as a monolayer culture in RPMI-1640 medium (Gibco), supplemented with 10% fetal calf serum (Gibco), 2 mM L-glutamine (Gibco), 200 units $l^{-1}$ insulin, and 1% penicillin/streptomycin (Gibco), at 37°C in air containing 5% carbon dioxide. The doubling time for an exponentially growing population of T-47D cells was 37.2 ± 2 h (Stokke et al, 1993). The human osteosarcoma cell line SAOS-2 was grown as monolayer culture in RPMI-1640 medium (Gibco), supplemented with 10% fetal calf serum (Gibco), 2 mM L-glutamine (Gibco), and 1% penicillin/streptomycin (Gibco), at 37°C in air containing 5% carbon dioxide. The human cervical carcinoma cell line NHIK 3025 (Nordbye and Oftebro, 1969; Oftebro and Nordbye, 1969) was grown as a monolayer culture in E2a medium (Puck et al, 1957) containing 20% human serum (prepared in the laboratory), 10% horse serum (Gibco), 2 mM L-glutamine (Gibco), and 1% penicillin/streptomycin (Gibco), at 37°C in air supplied with 5% carbon dioxide. NHIK 3025 cells contain integrated HPV18 and express the HPV18 E7 oncoprotein (Figure 4). The median cell cycle duration for NHIK 3025 cells was 16.5 h (Åmellem et al, 1994). All cell lines were kept in exponential growth by reculturing three times a week.

### Hypoxic cell cultures

The cells were seeded in 70-mm glass dishes (Anumbra, Czech Republic) 1 day before the experiment and incubated in a carbon dioxide incubator. At the appropriate time, the glass dishes were brought from the carbon dioxide incubator into a walk-in incubator room at 37°C. The medium content in each dish was reduced from 10 to 3.5 ml and placed without lids in a stainless steel chamber. Deoxygenation took place by continuous flushing of the chamber with a gas mixture (Hydro Gas, Norway) of highly purified 97% nitrogen, 3% carbon dioxide, and 100 or 1300 p.p.m. oxygen at 37°C, as described previously (Løvhaug et al, 1977). The hypoxic atmosphere in the chamber was established about 12 min after the start of flushing. Untreated control populations were kept in the carbon dioxide incubator during the experiment.

### Extraction, fixation and staining for measurement of nuclear-bound pRB and DNA content

All steps were carried out at 0°C. Harvested cells were washed once in phosphate-buffered saline (PBS). Detergent-extracted fixed cells were prepared by resuspending cells in 1.5 ml of low-salt detergent buffer [10 mM sodium chloride, 5 mM magnesium chloride, 0.1 mM phenylmethylsulphonyl fluoride, 0.1% Nonidet P-40, 10 mM phosphate buffer (pH 7.4)]. After 15 min, the extracted cells, which will be termed nuclei, were added to 0.5 ml of 4% paraformaldehyde under vortexing. Nuclei were fixed for 1 h, and then washed twice in washing buffer [10 mM Tris, 0.15 mM sodium chloride, 2 mM magnesium chloride and 0.1% Triton X-100 (pH 7.4)]. pRB was detected using the PMG3-245 monoclonal antibody (Pharmingen), which recognized both the under- and hyperphosphorylated forms of the protein (Mittnacht and Weinberg, 1991; Dowdy et al, 1993; Stokke et al, 1993). pRB staining was accomplished by a three-layer procedure. Nuclei were resuspended in 25 µl of 5% non-fat dry milk dissolved in washing buffer. Ten minutes later the anti-pRB monoclonal antibody was added to a final concentration of 2 µg $ml^{-1}$ in a total of 50 µl. The control samples received PBS and no primary antibody (data not shown). After a 30-min incubation, the nuclei were washed twice and incubated for 30 min in 100 µl of biotinylated horse anti-mouse $IgG_1$ (HAM) (Vector) diluted 1:50 in washing buffer. The nuclei were washed twice and then incubated for 30 min in 100 µl of streptavidin-FITC (Amersham) diluted 1:50 in washing buffer. The antibodies and the streptavidin-FITC were present in saturating amounts. After washing the nuclei twice, they were resuspended in 2 µg $ml^{-1}$ Hoechst 33258 for additional staining of DNA.

### Fixation and staining for BrdUrd and DNA content

Pulse-chase labelling with bromodeoxyuridine (BrdUrd) was used to record DNA synthesis in cells under hypoxic conditions. Cells were incubated with medium supplemented with 10 µM BrdUrd for 30 min and then washed twice in Hanks' salt solution and twice in medium before the hypoxic treatment. Harvested cells were washed once with PBS, fixed in 70% methanol and stored at –20°C. Fixed

© Cancer Research Campaign 1998

**Figure 1** Effect of moderate hypoxia on the phosphorylation state of pRB. T-47D cells were exposed to aerobic (lane 1) or moderately hypoxic conditions (1300 p.p.m. oxygen) (lanes 2 and 3) for 20 h. Cell populations were supplemented with medium containing 0.1 mM deoxycytidine (dC) during hypoxia (lane 3). Whole-cell extracts were prepared from T-47D cells. An equal amount of whole-cell protein extracts was loaded for each lane and separated by PAGE (8%). Western blot analysis was performed as described in Materials and methods. The blot was hybridized with anti-human pRB monoclonal antibody (Pharmingen, G3-245). One representative of two reproducible experiments is shown cells were washed with PBS, resuspended in 2 ml of 0.2% pepsin in 2 N hydrochloric acid, and incubated for 1 h at room temperature. From here all steps were performed at 0°C. The cells were washed three times in PBS, and a three-layer procedure for staining BrdUrd was used. Cells were resuspended in 50 μl of anti-BrdUrd antibody (Becton–Dickinson) diluted 1:10 in PBS containing 0.5% Tween 20 and 0.5% bovine serum albumin (BSA). After a 30 min incubation, cells were washed three times in PBS, and incubated with 50 μl of HAMs diluted 1:50 in PBS containing 0.5% Tween 20 and 0.5% BSA for 30 min. The cells were then washed three times in PBS and incubated with 50 μl of streptavidin-FITC diluted 1:50 in PBS containing 0.5% Tween 20 and 0.5% BSA for 30 min. After the cells were washed, they were resuspended in 2 μg $ml^{-1}$ Hoechst 33258 for additional staining of DNA.

**Figure 2** The cell cycle distribution, and the fraction of pRB+ nuclei, of exponentially growing cells under aerobic (**A–G**) or moderately hypoxic conditions (**H–O**). T-47D cells were exposed to moderately hypoxic conditions (1300 p.p.m. oxygen) for 20 h supplemented with plain medium (**H–K**) or medium containing 0.1 mM deoxycytidine (dC) (**L–O**). Control cells were labelled with BrdUrd for 30 min under aerobic conditions, washed, and either fixed immediately (**A–C**) or 20 h after labelling (**E–G**). Cells were treated with low salt detergent buffer for 15 min to isolate nuclei before addition of anti-pRB monoclonal antibody (Pharmingen, PMG 3-245) as described under Materials and methods (**D, H, L**). The fraction of pRB+ nuclei is indicated by the windows shown in histogram **D, H** and **L**. pRB (**D, H, L**) and BrdUrd-labelled cells (**A, E, I, M**) were FITC-stained with a three-layer procedure. DNA were stained with Hoechst 33258 and measured by flow cytometry as described in Materials and methods (**A–O**). The histograms in each figure (**A–O**) were generated from list-mode files by gating as described in Materials and methods. The various histograms show pRB content vs (abscissa) DNA content (ordinate) (**D, H, L**), BrdUrd content (abscissa) vs DNA content (ordinate) (**A, E, I, M**), and the single parameter DNA histograms from the BrdUrd− (**B, F, J, N**) and BrdUrd+ (**C, G, K, O**) fractions as indicated by the windows shown in histogram **A, E, I,** and **M**

 © Cancer Research Campaign 1998

**Figure 3** The fraction of cells in $G_1$, S and $G_2$ exposed to moderate hypoxia and the corresponding fraction of $pRB^+$ nuclei in the various cell cycle phases. T-47D cells were exposed to aerobic or moderately hypoxic conditions (1300 p.p.m. oxygen) for 20 h. Some cell populations were supplemented with medium containing 0.1 mM deoxycytidine (dC) during moderate hypoxia. The data were generated from the flow cytometric histograms shown in Figure 2. **A** shows the fraction of $G_1$-nuclei that are $pRB^+$ (○) and the fraction of cells in $G_1$ (●). **B** shows the fraction of S nuclei that are $pRB^+$ (□) and the fraction of cells in S (■). **C** shows the fraction of $G_2$+M nuclei that are $pRB^+$ (△) and the fraction of cells in G2+M (▲)

### Flow cytometry

Stained cells or nuclei were measured in a FACStar$^{plus}$ flow cytometer (Becton–Dickinson) equipped with two argon lasers (Spectra Physics) tuned to 488 nm and UV respectively. The following parameters were measured: forward light scatter (FSC), side scatter (SSC), FITC fluorescence intensity (pRB or BrdUrd), integrated Hoechst 33258 intensity (DNA content), Hoechst 33258 fluorescence pulse height, and Hoechst 33258 fluorescence pulse width. The FITC fluorescence intensity voltage (amplification) was kept constant at 500 V in all experiments. The data were gated on FSC vs SSC and Hoechst 33258 fluorescence pulse height vs pulse width to exclude dead cells and aggregates of cells respectively (not shown in the figures).

### Estimation of kinetic parameters

The fraction of $pRB^+$ (denoted bound pRB, which means that pRB is under-phosphorylated) in nuclei or cells with incorporated BrdUrd ($BrdUrd^+$ cells) was obtained by measuring the relative numbers of nuclei or cells in the peaks with high FITC fluorescence intensity values. The cycle distribution was estimated by computer simulation of the DNA distribution using the computer program Modfit.

### Sodium dodecyl sulphate (SDS)-polyacrylamide gel electrophoresis and Western blotting

For protein analysis by polyacrylamide gel electrophoresis (PAGE), cells attached to glass dishes were washed once in ice-cold PBS and then scraped and dissolved in 100 μl of sample buffer (Laemmli, 1970), containing 0.1 mM phenylmethylsulphonyl fluoride, 25 μg $ml^{-1}$ aprotinin, 24 μM leupeptin and 100 nM okadaic acid on ice. The whole-cell extracts were heated in boiling water for 5 min. Equal amounts of whole-cell protein extracts were loaded in each lane and separated by electrophoresis in 8% discontinuous SDS–polyacrylamide gels (Laemmli, 1970), with a 4% stacking gel. Proteins were transferred onto nitrocellulose membrane (NitroPure, MSI) using an electroblotter in 25 mM Tris-HCl (pH 8.3), 150 mM glycine and 15% methanol. The membranes were then immunolabelled with 2 μg $ml^{-1}$ anti-human monoclonal antibody against pRB (Pharmingen, PMG3-245), $p^{16INK4a}$ (Pharmingen, G175-405) or $p21^{WAF1}$ (Oncogene Science, EA10), and detected with Amersham ECL Western Detection kit (Amersham). The manufacturer's protocol was slightly modified with the additions of 5% non-fat dry milk and 0.02% Triton X-100 added to the staining solution.

### Reverse transcriptase polymerase chain reaction (RT-PCR) analysis

RT-PCR was used to check for appropriate expression of the HPV18 E7 gene in NHIK 3025 cells. mRNA was isolated from cell lysates and subjected to a second cycle of purification before applying RT-PCR (Boehringer Mannheim mRNA isolation kit). Reverse transcription with random hexamers was performed on 0.05 μg of mRNA with 1.25 U reverse transcriptase in a 50-μl reaction (Perkin Elmer RNA PCR kit), followed by PCR reactions using 2.5 U *Taq* DNA polymerase (Boehringer Mannheim) in a 50-μl reaction. The upper primer, p1E7 (CCG AGC ACG ACA GGA ACG ACT), from position 533 to 553, and the lower primer, p2E7 (TCG TTT TCT TCC TCT GAG TCG CTT), from position 682 to 705, were used as described previously (Hagmar et al, 1992). Both these primers were located within the E7 gene. Another lower primer, p3E4 (GGA ATA CGG TGA GGG GGT GTG), from position 3483 to 3503 within the E4 gene was used with the same upper primer (p1E7) in separate reactions (see Figure 4A). The reverse transcriptase was omitted in the control reactions to confirm the absence of DNA contamination in the reaction tubes. The PCR products were analysed on 3% NuSieve GTG agarose gels (FCM) and stained with ethidium bromide.

### RESULTS

### Addition of pyrimidine deoxynucleosides do not overcome the inhibition of DNA synthesis induced by moderate hypoxia in cells expressing wild-type pRB

In T-47D cells grown under aerobic conditions, the pRB retained in the nuclei was predominantly in its phosphorylated form (Figure 1, lane 1), which is confirmed by the high fraction of $pRB^-$ nuclei measured by flow cytometry (Figure 2D). However, a shift in the phosphorylation state of pRB to its under-phosphorylated form is observed after 20 h growth under moderately hypoxic conditions (1300 p.p.m. oxygen) (Figure 1, lane 2). The dephosphorylation of pRB is not affected by the absence or presence of 0.1 mM dC under hypoxic conditions (Figure 1, lane 3).

To relate the effect variations of nuclear binding of pRB ($pRB^+$) during moderate hypoxia to the cell cycle stage, the fraction of $pRB^+$ nuclei were generated from flow cytometric list mode data for nuclei derived from cells in $G_1$, S and $G_2$ phases of the cell cycle. For aerobic (i.e. control) T-47D cells, $pRB^+$ nuclei were found in the $G_1$ phase only (Figure 2D). A dramatic change in the fraction of $pRB^+$ nuclei was observed after 20 h exposure to moderate hypoxia (1300 p.p.m. oxygen) (Figure 2 h). The most pronounced increase in the fraction of $pRB^+$ nuclei (dephosphorylation of pRB) occurred for T-47D cells in S and $G_2$, whereas only a modest increase was observed in the fraction of $pRB^+$ nuclei in $G_1$ (Figure 2 h).

© Cancer Research Campaign 1998

**Figure 4** RT-PCR amplification of HPV18 gene expression. (**A**) Diagram of HPV18 region analysed. (**B**) mRNA was isolated from NHIK 3025 (lanes 2–4), HeLa S3 (lanes 5–7) and MCF-7 cells (lane 8). Lane 1 represents 100 bp DNA ladder standard (Pharmacia). The same upper primer, p1E7 (bp 533–553), was included in all reactions (lanes 2–8). Two different lower primers were used. The p2E7 lower primer (bp 618–705) was used in the reactions shown in lanes 3, 4, 6, 7 and 8. The p3E4 lower primer (bp 3483–3503) is used in the reactions shown in lanes 2 and 5. The yield is two different transcripts, E7 (lanes 2 and 5) and E7^E4 (lanes 3 and 6), of 173 and 430 bp respectively. Reverse transcriptase was excluded from the reactions shown in lanes 4 and 7. MCF-7 cells were included as a negative control (lane 8) and HeLa S3 cells (lanes 5–7) as a positive control as the latter cells contain HPV18 (Boshart et al, 1984). Agarose gel electrophoresis of DNA from the RT-PCR amplification of the HPV18 probes was visualized by staining with ethidium bromide

In a previous study, we showed that addition of deoxycytidine during moderate hypoxia in NHIK 3025 cells reversed the cell cycle-inhibitory effect of hypoxia (Åmellem et al, 1994). In the present study, we therefore added 0.1 mM deoxycytidine to see if this treatment could reverse the hypoxia-induced arrest that was related to the rebinding (dephosphorylation) of pRB to nuclear structures in cells in S-phase. The data show, however, that neither the increase in the fraction of $pRB^+$ nuclei nor the cell cycle progression itself are affected by the addition of deoxycytidine under moderate hypoxia (Figure 2L). These data support our observation made by Western blotting (Figure 1, lane 3), indicating that pRB becomes dephosphorylated under moderate hypoxia both in the absence and in the presence of pyrimidine deoxynucleosides.

To visualize the magnitude of the changes in the fraction of $pRB^+$ nuclei in various cell cycle phases of the T-47D cells as presented in Figure 2D, H, and L, the fraction of $pRB^+$ nuclei was analysed for each cell cycle phase, and the results are shown in Figure 3. The fraction of $pRB^+$ nuclei with S and $G_2$ DNA content increased from 0.05 to > 0.90 during 20 h exposure to moderate hypoxia (Figure 3B and 3C). In comparison, the increase in the fraction of $pRB^+$ nuclei with $G_1$ DNA content taking place during the same treatment was less pronounced (from 0.37 to 0.76, Figure 3A).

DNA-replicating cells incorporate BrdUrd into their DNA ($BrdUrd^+$), and are easily distinguishable from non-BrdUrd-labelled cells ($BrdUrd^-$) (Figure 2A). In a pulse-chase experiment, exponentially growing T-47D cells were labelled with BrdUrd before the hypoxic treatment. The fraction of $BrdUrd^+$ with S-phase DNA content remained unchanged if one compares the situation immediately after the pulse and before hypoxia (i.e. aerobic conditions, Figure 2A and 2C) and after 20 h exposure to 1300 p.p.m. oxygen (Figure 2I and K), which means that no T-47D cells have left S-phase under 20 h of moderately hypoxic conditions. In contrast, the fraction of $BrdUrd^+$ with S-phase DNA content reached $G_1$ of the subsequent cell cycle if one compares the situation immediately after the pulse (Figure 2A and C) with the situation after 20 h exposure to aerobic conditions (Figure 2E and G). The presence of deoxycytidine under moderate hypoxia had no effect on the fraction of $BrdUrd^+$ cells in S-phase (Figure 2M and O), indicating that the addition of pyrimidine deoxynucleosides did not abolish the hypoxia-induced S-phase arrest. It was also evident from the fraction of $BrdUrd^-$ cells present, that, in contrast to the situation seen under aerobic conditions (Figure 2E and F), no T-47D cells enter S-phase under these hypoxic conditions (Figure 2J and N). Only in the $G_2$ compartment was a small decrease observed in the fraction of $BrdUrd^-$ cells after moderate hypoxia (compare Figure 2A with I or M), showing that cell division takes place under moderate hypoxia in cells with functional pRB.

### Addition of pyrimidine deoxynucleosides overcome the inhibition of DNA synthesis induced by moderate hypoxia in cells expressing the HPV18 E7 oncoprotein

The cervical carcinoma cell line, NHIK 3025, does not express pRB, as shown by Western blotting (Stokke et al, 1993) and flow cytometry (data not shown). RT-PCR was used to identify the expression of the HPV18 E7 gene in NHIK 3025 cells (Figure 4B, lanes 2 and 3). One upper primer in combination with either one of two lower primers from different parts of the HPV18 genome yield two products, confirming the expression of the E7 and the E7^E4 type transcripts (173 and 430 bp respectively). The RT-PCR was performed using one negative control (MCF-7 cells) (Figure 4b, lane 8), one positive HPV18 control from HeLa S3 cells (Figure 4b, lanes 5 and 6), and two reaction controls in which reverse transcriptase was excluded to ensure the absence of DNA contamination in the reaction tubes (Figure 4B, lanes 4 and 7). The HPV18 E7 oncoprotein has the ability to bind and thus inactivate pRB (Tommasino and Crawford, 1995). To induce a similar degree of cell cycle inhibition in NHIK 3025 cells as induced by 1300 p.p.m. oxygen in T-47D cells, we had to reduce the concentration to only 100 p.p.m. oxygen.

In a pulse-chase experiment, exponentially growing NHIK 3025 cells were labelled with BrdUrd before the hypoxic treatment. More than half the population of cells, initially labelled with BrdUrd ($BrdUrd^+$) (Figure 5C), remained in S-phase after 18 h exposure to 100 p.p.m. oxygen (compare Figure 5C with I), whereas the rest of the labelled cells reached either $G_2$+M (18%) or divided and reached $G_1$ (29%) of the subsequent cell cycle. It was also evident that only a few of the $BrdUrd^-$ NHIK 3025 cells, initially in $G_1$, entered S-phase under these hypoxic conditions (compare Figure 5B with H). Compared with the growth of control cells (i.e. under aerobic conditions) (Figure 5D–F), the hypoxic cells (Figure 5G–I), thus, reduced the rate of cell cycle progression significantly. However, the addition of 0.1 mM deoxycytidine under moderate hypoxia increased the rate of cell cycle progression significantly (Figure 2J–L). About 88% of the cells initially labelled with BrdUrd progressed out of S-phase in the presence of 0.1 mM deoxycytidine under moderately hypoxic conditions (compare Figure 5A with J or Figure 5C with L). This indicates that the addition of pyrimidine deoxynucleosides abolishes the S-phase arrest induced by moderate hypoxia (compare Figure 5G with J or Figure 5I

© Cancer Research Campaign 1998

**Figure 5** Flow cytometric histograms of BrdUrd content vs DNA content, and the cell cycle distribution of BrdUrd⁻ or BrdUrd⁺, respectively, of NHIK 3025 cells. Cells were exposed to aerobic (**A–F**) or moderately hypoxic conditions (100 p.p.m. oxygen) (**G–L**) for 18 h. The cells were labelled with BrdUrd for 30 min under aerobic conditions, washed and fixed either immediately (**A–C**), 18 h after exposure to aerobic conditions ( **D–F**) or 18 h after exposure to moderately hypoxic conditions (**G–L**). Cell populations were supplemented with medium containing 0.1 mM deoxycytidine (dC) during hypoxia (**J–L**). BrdUrd-labelled cells were FITC-stained with a three-layer procedure, whereas DNA were stained with Hoechst 33258 and measured by flow cytometry as described in Materials and methods. The histograms show BrdUrd content (abscissa) vs DNA content (ordinate) (**A, D, G, J**), and the DNA histograms from the BrdUrd⁻ (**B, E, H, K**) and BrdUrd⁺ (**C, F, I, L**) fractions as indicated by the windows shown in histograms **A, D, G**, and **J**

with L) in cells expressing the HPV18 E7 oncoprotein. The presence of deoxycytidine also stimulated BrdUrd⁻ cells in $G_1$ to progress further into S and $G_2$+M phase under moderately hypoxic conditions (compare Figure 5G with J or Figure 5H with K).

### Addition of pyrimidine deoxynucleosides overcome the inhibition of DNA synthesis induced by moderate hypoxia in cells which lack pRB expression

To further determine whether the hypoxia-induced cell cycle arrest in pRB-deficient cells is due to lack of pyrimidine deoxynucleosides, we exposed SAOS-2 cells to 1300 p.p.m. oxygen for 18 h in the absence or presence of 0.1 mM deoxycytidine (Figure 6). SAOS-2 cells lack functional pRB (Ewen et al, 1993). Exponentially growing SAOS-2 cells were pulse-labelled with BrdUrd before the hypoxic treatment. We found that more than half the population of cells, initially labelled with BrdUrd (BrdUrd⁺) (Figure 6A), remained in S-phase after 20 h exposure to 1300 p.p.m. oxygen (Figure 6C). However, most of these BrdUrd⁺ cells arrested in S-phase under moderate hypoxia were able to complete DNA synthesis in the presence of 0.1 mM deoxycytidine (compare Figure 6C with D, Figure 7D). Even in the presence of deoxycytidine the rate of cell cycle progression was still reduced under moderately hypoxic compared with under aerobic conditions (compare Figure 6B with D). Some of the cells initially in $G_1$ (BrdUrd⁻) entered S-phase under these hypoxic conditions (compare Figure 6A with C). These results support the experiment presented in Figure 5, suggesting that in the absence of functional pRB inhibition of de novo synthesis of pyrimidine deoxynucleotides is the main mechanism responsible for the hypoxia-induced arrest in S-phase.

Analysis of the DNA distribution in the BrdUrd⁻ and the BrdUrd⁺ fraction in both T-47D (Figure 2), NHIK 3025 cells (Figure 5) and SAOS-2 cells (Figure 6) are presented in Figure 7.

© Cancer Research Campaign 1998

**Figure 6** Flow cytometric contour plots of BrdUrd content vs DNA content of SAOS-2 cells. Cells were exposed to aerobic (**A** and **B**) or moderately hypoxic conditions (1300 p.p.m. oxygen) (**C** and **D**) for 20 h. The cells were labelled with BrdUrd for 30 min under aerobic conditions, washed and fixed either immediately (**A**), 20 h after exposure to aerobic conditions (**B**) or 20 h after exposure to moderately hypoxic conditions (**C** and **D**). Cell populations were supplemented with medium containing 0.1 mM deoxycytidine (dC) during hypoxia (**D**). BrdUrd-labelled cells were FITC-stained with a three-layer procedure, whereas DNA were stained with Hoechst 33258 and measured by flow cytometry as described in Materials and methods.

**Figure 7** The fraction of BrdUrd⁻ cells and BrdUrd⁺ cells, respectively, in $G_1$, S and $G_2$+M after exposure to moderate hypoxia. T-47D cells (●) and SAOS-2 cells (□) were exposed to aerobic or moderately hypoxic conditions (1300 p.p.m. oxygen) for 20 h, whereas NHIK 3025 cells (○) were exposed to aerobic or moderately hypoxic conditions (100 p.p.m. oxygen) for 18 h, both in the absence or in the presence of medium supplemented with 0.1 mM dC under moderately hypoxic conditions. The cells were labelled with BrdUrd for 30 min as described in Materials and methods. The data were generated from the flow cytometric histograms shown in Figures 2, 5 and 6. The fraction of BrdUrd⁺ cells are shown in **B** ($G_1$ phase DNA content), **D** (S-phase DNA content) and **F** ($G_2$+M phase DNA content). The fraction of BrdUrd⁻ cells is shown in **A** ($G_1$ phase DNA content), **C** (S-phase DNA content) and **E** ($G_2$+M phase DNA content)

### Cdk inhibitors are not induced in p53-deficient cells under hypoxic conditions

We next asked whether this hypoxia-induced cell cycle arrest could be due to activation of cdk inhibitory proteins. However, $p16^{INK4a}$ was not detected by Western blotting in T-47D cells whether the cells were cultured under aerobic or moderately hypoxic conditions (Figure 8, lanes 3 and 4 respectively). In contrast, pRB-deficient NHIK 3025 cells expressed high levels of $p16^{INK4a}$ under both aerobic conditions and after 20 h exposure to moderately hypoxic conditions (Figure 8, lanes 1 and 2 respectively).

As T-47D cells express mutated p53 (Bartek et al, 1990), and the wild-type p53 functions is abrogated by the presence of HPV E6 in NHIK 3025 cells, the induction of $p21^{WAF1/CIP1}$ must occur independently of p53. Both NHIK 3025 and T-47D cells express low levels of $p21^{WAF1/CIP1}$ under aerobic conditions (Figure 8, lanes 1 and 3 respectively). However, $p21^{WAF1/CIP1}$ decreased below the detection level in both these cell types after exposure to moderately hypoxic conditions for 20 h (Figure 8, lanes 2 and 4 respectively).

### DISCUSSION

### Cell cycle regulation during moderate hypoxia in cells with functional or non-functional pRB

The results presented here demonstrate that pRB is dephosphorylated (Figure 1) and re-bound (here termed pRB⁺) (Figure 2H and L) in the nucleus in T-47D cells under moderate hypoxia (1300 p.p.m. oxygen). This marked change in the state of pRB phosphorylation towards a more under-phosphorylated form under moderately hypoxic conditions is most pronounced in the nucleus of cells in the S and $G_2$ phases of the cell cycle. In more than 90% of these cells, pRB is dephosphorylated and re-bound in the nucleus during a 20 h treatment with moderate hypoxia, which is opposite to the situation under aerobic conditions, in which only a few per cent of cells in S and $G_2$ are pRB⁺. No T-47D cells entered S-phase, and no progression through S-phase was observed, during the 20 h exposure to 1300 p.p.m. oxygen (Figure 2). The slow dephosphorylation of pRB, as observed under extremely hypoxic conditions (Åmellem et al, 1996), indicates that the immediate arrest is due to mechanisms other than pRB. The slow dephosphorylation of pRB, however, can explain why cells that are in $G_2$ at the onset of hypoxia complete the cell cycle and enter $G_1$ of the next cell cycle during hypoxia (Figure 2). Hypoxic stress, which becomes increasingly toxic with time, particularly to cells in S-phase (Merz and Schneider, 1983; Spiro et al, 1984; Åmellem and Pettersen, 1991*a*), may activate pRB to prevent harmful effects that could follow from continued DNA replication during hypoxia.

© Cancer Research Campaign 1998

**Figure 8** Effect of moderate hypoxia on the p16$^{INK4a}$ and p21$^{WAF1/CIP1}$ protein level. NHIK 3025 and T-47D cells were exposed to aerobic (lanes 1 and 3 respectively) or moderately hypoxic conditions (1300 p.p.m. oxygen) (lanes 2 and 4 respectively) for 20 h. The samples were prepared from whole-cell lysates. An equal amount of protein was loaded for each lane and separated by PAGE (8%). The relative p16$^{INK4a}$ and p21$^{WAF1/CIP1}$ protein level was determined by Western blot analysis as described in Materials and methods. The blots were hybridized with anti-human p16$^{INK4a}$ monoclonal antibody (Pharmingen, G175-405) or anti-human p21$^{WAF1}$ monoclonal antibody (Oncogene Science, EA10). One representative of three reproducible experiments is shown

Several lines of evidence have linked the under-phosphorylated form of pRB to its role as an inhibitor of cell cycle progression through $G_1$ (reviewed by Weinberg, 1995). It is, therefore, tempting to suggest that dephosphorylation of pRB under moderate hypoxia is also responsible for the hypoxia-induced cell cycle arrest in S-phase. The hypoxia-induced dephosphorylation of pRB could be due to either a prevention of a kinase or an activation of a phosphatase or both. The growth-suppressing activity of pRB is down-regulated by various cdks (i.e. cdk4/6-cyclin D, cdk2-cyclin E and cdk2-cyclin A) whose kinase activity is negatively regulated by cdk inhibitors of the p16 and p21 families. As p21$^{WAF1/CIP1}$, and the related p27$^{Kip1}$, can associate with multiple cyclin–cdk complexes, associated with $G_1$ and S-phases (reviewed by Sherr and Roberts, 1995), it is most likely that they can exert controls at multiple points in the cell cycle. A possible explanation to the dephosphorylation of pRB during hypoxia could, therefore, be that it is mediated by p53-dependent activation of p21$^{WAF1/CIP1}$. However, the p53 gene is mutated in T-47D cells (Nigro et al, 1989), and the p53 protein level does not change in response to neither hypoxia (Åmellem et al, 1997) nor irradiation (data not shown), indicating that p53 is not involved in the possible inhibition of pRB kinases during hypoxia. This supports the view that dephosphorylation of pRB is due to activation of a pRB phosphatase in the absence of constitutive pRB kinase activity. This view is further supported by our previous results, showing that dephosphorylation of pRB under extremely hypoxic conditions is independent of the p53 gene status (Åmellem et al, 1996). It has been suggested that pRB-mediated $G_1$ arrest, independent of p53, could be achieved through activation of a pRB phosphatase (Dou et al, 1995; An and Dou, 1996). On the other hand, p21$^{WAF1/CIP1}$ can also be activated by p53-independent mechanisms (Macleod et al, 1995; Russo et al, 1995). Our results show that both T-47D and NHIK 3025 cells express low levels of p21$^{WAF1/CIP1}$ under aerobic conditions. T-47D cells have previously been shown to express barely detectable levels of p21$^{WAF1/CIP1}$ mRNA (Sheikh et al, 1994). However, p21$^{WAF1/CIP1}$ was not detectable by Western blot analysis under moderately hypoxic conditions in both these cell types. In addition, p16$^{INK4a}$ was not detectable in T-47D cells, whether the cells were cultured under aerobic or moderately hypoxic conditions. Furthermore, two other members of the p16 family, p15$^{INK4b}$ and p18$^{INK4c}$, are found to be mutated in T-47D cells (Zariwala et al, 1996). Taken together, these data suggest that neither members of the p16 family nor p21$^{WAF1/CIP1}$ are involved in the hypoxia-induced cell cycle arrest in p53-deficient T-47D cells. The observed dephosphorylation of pRB under moderate hypoxia did not depend on the activation of cdk inhibitors, supporting the suggestion above that dephosphorylation is due to activation of a pRB phosphatase. Furthermore, hypoxia has been shown to induce p21$^{WAF1/CIP1}$, but only in a wild-type p53-dependent manner (Graeber et al, 1994). In general, the p53-dependent expression of p21$^{WAF1/CIP1}$ has mainly been associated with the induction of cell cycle arrest (reviewed by Sherr and Roberts, 1995). However, recent studies have suggested that the role of p53 under hypoxic conditions is not associated with cell cycle arrest (Graeber et al, 1994), but merely with the induction of apoptosis (Graeber et al, 1996; Åmellem et al, 1997). Thus, the function of p21$^{WAF1/CIP1}$ under hypoxic conditions remains unknown.

In contrast to T-47D cells, the human cervical carcinoma NHIK 3025 cells express the HPV18 E7 oncoprotein (Figure 4), known to bind pRB and thereby inhibit its normal function. We were unable to detect any pRB in NHIK 3025 cells as measured by flow cytometry (data not shown) and Western blotting (Stokke et al, 1993). However, this pRB-deficient cell line express high levels of p16$^{INK4a}$ under both aerobic and hypoxic conditions. A high level of p16$^{INK4a}$ has been shown previously in other cervical carcinoma cell lines (Parry et al, 1995). Overexpression of p16$^{INK4a}$ has been reported to prevent proliferation in pRB-functional cells, but is ineffective in pRB-deficient cells (Guan et al, 1994). Therefore, in NHIK 3025 cells lacking functional pRB, the inhibitory function of p16$^{INK4a}$ may not be manifested.

The oxygen sensitivity regarding cell proliferation is rather different in T-47D cells compared with NHIK 3025 cells. No T-47D cells in $G_1$ seem to enter S-phase during the 20-h exposure to 1300 p.p.m. oxygen, despite the fact that approximately 25% of the $G_1$ cells are still pRB$^-$. This could indicate that mechanisms other than pRB, such as the oxygen-dependent restriction in $G_1$ (Åmellem et al, 1994), are active in these cells, even under moderate hypoxia, and prevent them from leaving $G_1$. This is in contrast to the situation in NHIK 3025 cells where the oxygen concentration has to be reduced below 20 p.p.m. to completely inhibit entry into S-phase (Pettersen and Lindmo, 1983). Thus, in the present experiments with NHIK 3025 we used 100 p.p.m. oxygen in contrast to 1300 p.p.m. oxygen used on T-47D cells. Still, NHIK 3025 cells are able to proliferate under such conditions, although the rate of progression is highly reduced (Figure 5). Although the normal dNTP pools are in general sufficient for only a few minutes of DNA synthesis (Reichard, 1988), some NHIK 3025 cells are still able to progress out of S-phase and even further into the next cell cycle (Figure 5). This indicates that the oxygen-dependent DNA precursor metabolism continues to be active for a few hours even at oxygen concentrations as low as 100 p.p.m. before the NHIK 3025 cells become arrested.

The E7 oncoprotein has been shown to disrupt the interaction between the transcription factor E2F and pRB, and this protein complex is absent in various cervical carcinoma cell lines that express E7 (Chellappan et al, 1992). This protein complex is believed to result in the growth suppressive function that pRB exerts in the early parts of $G_1$ (Bagchi et al, 1991; Helin et al, 1993; Lees et al, 1993). E7 binds only to the under-phosphorylated form of pRB (Dyson et al, 1989), which is its functional form. By doing so, E7 presumably promotes cells to proceed through the cell

© Cancer Research Campaign 1998

cycle. Indeed, expression of the E7 oncoprotein allows cell cycle progression, both under hypoxic conditions (Figure 5) and after DNA damage induced by irradiation (Hickman et al, 1994; Slebos et al, 1994).

### The role of pyrimidine metabolism in the regulation of cell proliferation under moderate hypoxia in cells with functional or non-functional pRB

Addition of deoxynucleosides to T-47D cells during exposure to moderate hypoxia did not counteract the hypoxia-induced cell cycle arrest in these cells (Figures 1 and 2). Similar lack of response has also been found in other cell types with normal pRB status, such as MCF-7 and Reh cells (data not shown). We can rule out the possible explanation that hypoxia might have induced some perturbation of the salvage pathway, as the thymidine analogue BrdUrd was readily incorporated into DNA. Instead, it is more likely that cell cycle inhibition in T-47D cells under protracted moderate hypoxia is due to activation of pRB. This is highly supported by our observation that hypoxia-induced cell cycle arrest of NHIK 3025 cells (Figure 5) and SAOS-2 cells (Figure 6), both lacking functional pRB, was abolished by addition of pyrimidine deoxynucleosides (i.e. dC). It is also in accordance with this explanation that the addition of exogenously added deoxycytidine did not affect the phosphorylation state of pRB in T-47D cells under moderate hypoxia (Figure 1), i.e. pRB also remained dephosphorylated in the presence of excess amounts of deoxynucleosides. pRB, thus, seems to over-rule the stimulatory effect of addition of deoxynucleosides, and, therefore, may be the main regulator of cell proliferation under moderate hypoxia. One important aspect, here, is the slow kinetics regarding the hypoxia-induced dephosphorylation of pRB (Åmellem et al, 1996). This implies that the immediate arrest observed under moderate hypoxia in T-47D cells is mediated by some mechanisms other than through the pRB pathway. One possible explanation is that the oxygen-dependent catalytic activity of dihydroorotate dehydrogenase and/or ribonucleotide reductase in T-47D cells needs higher concentrations of oxygen than the concentrations needed in NHIK 3025 or SAOS-2 cells in order to be enzymaticaly functional. However, this remains to be elucidated.

The suppressive effects of pRB is supposed to be mediated by the underphosphorylated form of the protein (Buchkovich et al, 1989; DeCaprio et al, 1989; Mihara et al, 1989) by sequestering the transcription factor E2F1-3 (Bagchi et al, 1991; Chellappan et al, 1991). If, therefore, moderate hypoxia promotes rebinding of pRB to E2F-1, we can have a simple explanation of how pRB inhibit progression through S-phase during such hypoxia. Recently, it has been demonstrated that genes encoding S-phase-specific proteins are induced by E2F-1 [including both subunits of ribonucleotide reductase (R1 and R2), DNA polymerase α, thymidylate synthase, proliferating cell nuclear antigen] (DeGregori et al, 1995). In addition, E2F-1 has been shown to be a prerequisite for the expression of genes implicated in growth control (including *cdc*2, *cyclin* A, *cyclin* E, c-*myc*, B-*myb*, and the *E2F*-1 gene itself) that have E2F-binding sites in their promoters (reviewed by La Thangue, 1994). For example, cyclin A, which is involved in the control of cell cycle progression through S-phase, and at the entry into mitosis (Girard et al, 1991; Pagano et al, 1992), is reduced in hypoxia-arrested cells in S-phase (Ludlow et al, 1993*b*). Another possibility that might explain the involvement of pRB in hypoxia-induced growth arrest is the recent discovery that pRB can inhibit cell proliferation through a more global regulation of genes by repressing the transcription of all three classes of nuclear RNA polymerases (Cavanaugh et al, 1995; White et al, 1996). Thus, from the present as well as previous results, it seems that the hypoxia-induced cell cycle arrest is due to both a direct inhibition of DNA synthesis (Löffler, 1992; Åmellem et al, 1994) and by activation of pRB (Ludlow et al, 1993*b*; this study), a key member of the cell cycle checkpoint network, with E2F-1 as a possible link between them.

Addition of pyrimidine deoxynucleosides to NHIK 3025 and SAOS-2 cells partly reversed the S-phase arrest induced by moderate hypoxia. However, normal rate of cell cycle progression was not established, indicating that additional mechanisms regulate the rate of cell proliferation under such conditions. Similar effects have been observed in Ehrlich ascites cells (Löffler, 1992). Whether these cells contain wild-type pRB has, to our knowledge, not been investigated. From our own data on NHIK 3025 and SAOS-2 cells, we conclude as follows: in the absence of pRB checkpoint control, the oxygen-dependent de novo synthesis of pyrimidine deoxynucleotides seems to be the major limiting step in the control of cell cycle progression through S-phase under moderately hypoxic conditions.

It seems, from the present data, that the pRB status survey the physiological growth conditions of the cell, and that it may, thus, function as a stress indicator in the cell. This is, however, a slowly-acting regulation and even in cells with functional pRB the immediate arrest in S-phase after the onset of moderate hypoxia is believed to be a consequence of blocking de novo synthesis of pyrimidine deoxynucleotides due to inhibition of the two oxygen-dependent enzymes, dihydroorotate dehydrogenase and ribonucleotide reductase (Probst et al, 1989; Löffler, 1992; Åmellem et al, 1994; Brischwein et al, 1997). An additional explanation proposed by Probst et al (1988) suggests that hypoxia inhibits the initiation of new replicones, whereas DNA chain elongation and termination proceed during hypoxia. They further demonstrated that replicon initiation depends on one or several short-lived proteins that are also formed under hypoxic conditions, suggesting that hypoxic cells in S-phase are arrested in a state fully prepared for entering DNA replication (Riedinger et al, 1992).

The dephosphorylated form of pRB seem to take over as the main negative regulator about 4 h after the hypoxia-induced cell cycle arrest (Åmellem et al, 1996). Thus, the molecular down-regulation of DNA replication under moderate hypoxia is gradually overridden by a member of the cell cycle checkpoint network, pRB, which seems to serve as the main regulator of progression through S-phase under moderately hypoxic conditions.

### ACKNOWLEDGEMENTS

The skillful technical assistance of Charlotte Borka, Simen S. Reine and Torild Aasen is gratefully acknowledged. We are grateful to Frank Karlsen for the HPV primers. The present study was supported by the The Norwegian Radium Hospital Research Foundation and the Norwegian Cancer Society.

### ABBREVIATIONS

Ribonucleotide reductase [EC 1.17.4.1]; dihydroorotate dehydrogenase [EC 1.3.3.1].; dNTP, deoxynucleotide triphosphate; dC,

 © Cancer Research Campaign 1998

deoxycytidine; BrdUrd: bromodeoxyuridine; RT-PCR, reverse transcriptase polymerase chain reaction; HPV, human papillomavirus.

### REFERENCES

An B and Dou QP (1996) Cleavage of retinoblastoma protein during apoptosis: An interleukin 1b-converting enzyme-like protease as candidate. *Cancer Res* **56**: 438–442

Anderson GR and Farkas BK (1988) The major anoxic stress response protein p34 is a distinct lactate dehydrogenase. *Biochemistry* **27**: 2187–2192

Åmellem Ø and Pettersen EO (1991*a*) Cell inactivation and cell cycle inhibition as induced by extreme hypoxia: the possible role of cell cycle arrest as a protection against hypoxia-induced lethal damage. *Cell Prolif* **24**: 127–141

Åmellem Ø and Pettersen EO (1991*b*) The role of protein accumulation on the kinetics of entry into S phase following extreme hypoxia. *Anticancer Res* **11**: 1083–1088

Åmellem Ø, Löffler M and Pettersen EO (1994) Regulation of cell proliferation under extreme and moderate hypoxia: the role of pyrimidine (deoxy)nucleotides. *Br J Cancer* **70**: 857–866

Åmellem Ø, Stokke T, Sandvik JA and Pettersen EO (1996) The retinoblastoma gene product is reversibly dephosphorylated and bound in the nucleus in S and $G_2$ phase during hypoxic stress. *Exp Cell Res* **227**: 106–115

Åmellem Ø, Stokke T, Sandvik JA, Smedshammer L and Pettersen EO (1997) Hypoxia-induced apoptosis in human cells with normal p53 status and function, without any alteration in the nuclear protein level. *Exp Cell Res* **232**: 361–370

Bagchi S, Weinmann R and Raychaudhuri P (1991) The retinoblastoma protein copurifies with E2F-I, an E1A-regulated inhibitor of the transcription factor E2F. *Cell* **65**: 1063–1072

Bartek J, Iggo R, Gannon J and Lane DP (1990) Genetic and immunochemical analysis of mutant p53 in human breast cancer cell lines. *Oncogene* **5**: 893–899

Boshart M, Gissmann L, Ikenberg H, Kleinheinz A, Scheurlen W and Zur Hausen H (1984) A new type of papillomavirus DNA, its presence in genital cancer biopsies and in cell lines derived from cervical cancer. *EMBO J* **3**: 1151–1157

Brischwein K, Engelcke M, Riedinger H-J and Probst H (1997) Role of ribonucleotide reductase and deoxynucleotide pools in the oxygen-dependent control of DNA replication in Ehrlich ascites cells. *Eur J Biochem* **244**: 286–293

Buchkovich K, Duffy LA and Harlow E (1989) The retinoblastoma protein is phosphorylated during specific phases of the cell cycle. *Cell* **58**: 1097–1105

Cavanaugh AH, Hempel WM, Taylor LJ, Rogalsky V, Todorov G and Rothblum LI (1995) Activity of RNA polymerase I transcription factor UBF blocked by *Rb* gene product. *Nature* **374**: 177–180

Chellappan SP, Hiebert S, Mudryj M, Horowitz JM and Nevins JR (1991) The E2F transcription factor is a cellular target for the RB protein. *Cell* **65**: 1053–1061

Chellappan SP, Kraus VB, Kroger B, Munger K, Howley PM, Phelps WC and Nevins JR (1992) Adenovirus E1A, simian virus 40 tumour antigen, and human papillomavirus E7 protein share the capacity to disrupt the interaction between transcription factor E2F and the retinoblastoma gene product. *Proc Natl Acad Sci USA* **89**: 4549–4553

Chen PL, Scully P, Shew JY, Wang JY and Lee WH (1989) Phosphorylation of the retinoblastoma gene product is modulated during the cell cycle and cellular differentiation. *Cell* **58**: 1193–1198

Decaprio JA, Ludlow JW, Lynch D, Furukawa Y, Griffin J, Piwnica-Worms H, Huang CM and Livingston DM (1989) The product of the retinoblastoma susceptibility gene has properties of a cell cycle regulatory element. *Cell* **58**: 1085–1095

DeGregori J, Kowalik T and Nevins JR (1995) Cellular targets for activation by the E2F1 transcription factor include DNA synthesis- and $G_1$/S-regulatory genes. *Mol Cell Biol* **15**: 4215–4224

Dou QP, An B and Will PL (1995) Induction of a retinoblastoma phosphatase activity by anticancer drugs accompanies p53-independent $G_1$ arrest and apoptosis. *Proc Natl Acad Sci USA* **92**: 9019–9023

Dowdy SF, Hinds PW, Louie K, Reed SI, Arnold A and Weinberg RA (1993) Physical interaction of the retinoblastoma protein with human D cyclins. *Cell* **73**: 499–511

Dyson N, Howley PM, Munger K and Harlow E (1989) The human papillomavirus-16 E7 oncoprotein is able to bind to the retinoblastoma gene product. *Science* **243**: 934–937

Ewen ME, Sluss HK, Sherr CJ, Matsushime H, Kato J and Livingston DM (1993) Functional interactions of the retinoblastoma protein with mammalian D-type cyclins. *Cell* **73**: 487–497

Girard F, Strausfeld U, Fernandez A and Lamb NJC (1991) Cyclin A is required for the onset of DNA replication in mammalian fibroblasts. *Cell* **67**: 1169–1179

Graeber TG, Peterson JF, Tsai M, Monica K, Fornace AJ Jr and Giaccia AJ (1994) Hypoxia induces accumulation of p53 protein, but activation of a $G_1$-phase checkpoint by low-oxygen conditions is independent of p53 status. *Mol Cell Biol* **14**: 6264–6277

Graeber TG, Osmanian C, Jacks T, Housman DE, Koch CJ, Lowe SW and Giaccia AJ (1996) Hypoxia-mediated selection of cells with diminished apoptotic potential in solid tumours. *Nature* **379**: 88–91

Guan K-L, Jenkins CW, Li Y, Nichols MA, Wu X, O'Keefe CL, Matera AG and Xiong Y (1994) Growth suppression by p18, a $p16^{INK4a/MTS1}$- and $p14^{INK4B/MTS2}$-related CDK6 inhibitor, correlates with wild-type pRb function. *Genes Dev* **8**: 2939–2952

Hagmar B, Johansson B, Kalantari M, Petersson Z, Skyldberg B and Walaas L (1992) The incidence of HPV in a Swedish series of invasive cervical carcinoma. *Med Oncol Tumour Pharmacother* **9**: 113–117

Heacock CS and Sutherland RM (1990) Enhanced synthesis of stress proteins caused by hypoxia and relation to altered cell growth and metabolism. *Br J Cancer* **62**: 217–225

Helin K, Harlow E and Fattaey AR (1993) Inhibition of E2F-1 transactivation by direct binding of the retinoblastoma protein. *Mol Cell Biol* **13**: 6501–6508

Hickman ES, Picksley SM and Vousden KH (1994) Cells expressing HPV16 E7 continue cell cycle progression following DNA damage induced p53 activation. *Oncogene* **9**: 2177–2181

Hockel M, Knoop C, Schlenger K, Vorndran B, Baussmann E, Mitze M, Knapstein PG and Vaupel P (1993) Intratumoral $pO_2$ predicts survival in advanced cancer of the uterine cervix. *Radiother Oncol* **26**: 45–50

Keydar I, Chen L, Karby S and Weiss FR, Delarea J, Radu M, Chaitcik S and Brenner HJ (1979) Establishment and characterization of a cell cline of human breast carcinoma origin. *Eur J Cancer* **15**: 659–670

Koch CJ, Kruuv J, Frey HE and Snyder RA (1973) Plateau phase in growth induced by hypoxia. *Int J Radiat Biol* **23**: 67–74

La Thangue NB (1994) DRTF1/E2F: an expanding family of heterodimeric transcription factors implicated in cell cycle control. *Trends Biochem Sci* **19**: 108–113

Laemmli UK (1970) Cleavage of structural proteins during the assembly of the head of bacteriophage T4. *Nature* **227**: 680–685

Lam EW-F and La Thangue NB (1994) DP and E2F proteins: Coordinating transcription with cell cycle progression. *Curr Opin Cell Biol* **6**: 859–866

Lees JA, Saito M, Vidal M, Valentine M, Look T, Harlow E, Dyson N and Helin K (1993) The retinoblastoma protein binds to a family of E2F transcription factors. *Mol Cell Biol* **13**: 7813–7825

Löffler M (1992) A cytokinetic approach to determine the range of $O_2$-dependence of pyrimidine(deoxy)nucleotide biosynthesis relevant for cell proliferation. *Cell Prolif* **25**: 169–179

Löffler M, Postius S and Schneider F (1978) Anaerobiosis and oxygen recovery: Changes in cell cycle distribution of Ehrlich ascites tumor cells grown in vitro. *Virchow's Arch B Cell Path* **26**: 359–368

Löffler M, Schimpff-Weiland G and Follmann H (1983) Deoxycytidylate shortage is a cause of $G_1$ arrest of ascites tumor cells under oxygen deficiency. *FEBS Lett* **156**: 72–76

Ludlow JW, Glendening CL, Livingston DM and Decaprio JA (1993*a*) Specific enzymatic dephosphorylation of the retinoblastoma protein. *Mol Cell Biol* **13**: 367–372

Ludlow JW, Howell RL and Smith HC (1993*b*) Hypoxic stress induces reversible hypophosphorylation of pRB and reduction in cyclin A abundance independent of cell cycle progression. *Oncogene* **8**: 331–339

Løvhaug D, Wibe E, Oftebro R, Pettersen EO and Brustad T (1977) Recovery from X-ray induced damage in human cells grown in culture. *Neoplasma* **24**: 513–520

Macleod KF, Sherry N, Hannon G, Beach D, Tokino T, Kinzler K, Vogelstein B and Jacks T (1995) p53-dependent and independent expression of p21 during cell growth, differentiation, and DNA damage. *Genes Dev* **9**: 935–944

Merz R and Schneider F (1983) Growth characteristics of anaerobically treated early and late S-period of Ehrlich ascites tumor cells after reaeration. *Z Naturforsch* **38c**: 313–318

Mihara K, Cao X, Yen A, Chandler S, Driscoll B, Murphree AL, T'ang A and Fung YT (1989) Cell cycle-dependent regulation of phosphorylation of the human retinoblastoma gene product. *Science* **246**: 1300–1303

Mittnacht S and Weinberg RA (1991) $G_1$/S phosphorylation of the retinoblastoma protein is associated with an altered affinity for the nuclear compartment. *Cell* **65**: 381–393

© Cancer Research Campaign 1998

Nigro JM, Baker SJ, Preisinger AC, Jessup JM, Hostetter R, Cleary K, Bigner SH, Davidson N, Baylin S, Devilee P, Glover T, Collins FS, Weston A, Modali R, Harris CC and Vogelstein B (1989) Mutations in the p53 gene occur in diverse human tumour types. *Nature* **342**: 705–708

Nordbye K and Oftebro R (1969) Establishment of four new cell strains from human uterine cervix. *Exp Cell Res* **58**: 458

Oftebro R and Nordbye K (1969) Establishment of four new cell strains from human uterine cervix II. *Exp Cell Res* **58**: 459–460

Pagano M, Pepperkok R, Verde F, Ansorge W and Draetta G (1992) Cyclin A is required at two points in the human cell cycle. *EMBO J* **11**: 961–971

Parry D, Bates S, Mann DJ and Peters G (1995) Lack of cyclin D-Cdk complexes in Rb-negative cells correlates with high levels of $p16^{INK4a/MTS1}$ tumour suppressor gene product. *EMBO J* **14**: 503–511

Pettersen EO and Lindmo T (1983) Inhibition of cell cycle progression by acute treatment with various degrees of hypoxia: modifications induced by low concentrations of misonidazole present during hypoxia. *Br J Cancer* **48**: 809–817

Pettersen EO, Juul NO and Rønning ØW (1986) Regulation of protein metabolism of human cells during and after acute hypoxia. *Cancer Res* **46**: 4346–4351

Price BD and Calderwood SK (1992) Gadd45 and Gadd153 messenger RNA levels are increased during hypoxia and after exposure of cells to agents which elevate the levels of the glucose-regulated proteins. *Cancer Res* **52**: 3814–3817

Probst H, Schiffer H, Gekeler V, Kienzle-Pfeilsticker H, Stropp U, Stötzer K and Frenzel-Stötzer I (1988) Oxygen dependent regulation of DNA synthesis and growth of Ehrlich ascites tumor cells in vitro and in vivo. *Cancer Res* **48**: 2053–2060

Probst H, Schiffer H, Gekeler V and Scheffler K (1989) Oxygen dependent regulation of mammalian ribonucleotide reductase in vivo and possible significance for replicon initiation. *Biochem Biophys Res Commun* **163**: 334–340

Puck TT, Cieciura SJ and Fisher HW (1957) Clonal growth in vitro of human cells with fibroblastic morphology. *J Exp Med* **106**: 145–165

Reichard P (1988) Interactions between deoxyribonucleotide and DNA synthesis. *Annu Rev Biochem* **57**: 349–374

Riedinger H-J, Gekeler V and Probst H (1992) Reversible shutdown of replicon initiation by transient hypoxia in Ehrlich ascites cells. Dependence of initiation on short-lived protein. *Eur J Biochem* **210**: 389–398

Russo T, Zambrano N, Esposito F, Ammendola R, Cimino F, Fiscella M, Jackman J, O'Connor PM, Anderson CW and Appella E (1995) A p53-independent pathway for activation of WAF1/CIP1 expression following oxidative stress. *J Biol Chem* **270**: 29386–29391

Sciandra JJ, Subjeck JR and Hughes CS (1984) Induction of glucose-regulated proteins during anaerobic exposure and of heat-shock proteins after reoxygenation. *Proc Natl Acad Sci USA* **81**: 4843–4847

Sheikh MS, Li XS, Chen JC, Shao ZM, Ordonez JV and Fontana JA (1994) Mechanisms of regulation of WAF1/Cip1 gene expression in human breast carcinoma: role of p53-dependent and independent signal transduction pathways. *Oncogene* **9**: 3407–3415

Sherr, CJ (1994) The ins and outs of *RB*: Coupling gene expression to the cell cycle clock. *Trends Cell Biol* **4**: 15–18

Sherr CJ and Roberts JM (1995) Inhibitors of mammalian $G_1$ cyclin-dependent kinases. *Genes Dev* **9**: 1149–1163

Shi Y, Åmellem Ø and Pettersen EO (1993) Proteins specifically regulated under conditions of extreme hypoxia in human cells cultivated in vitro. *APMIS* **101**: 75–82

Slebos RJC, Lee MH, Plunkett BS, Kessis TD, Williams BO, Jacks T, Hedrick L, Kastan MB and Cho KR (1994) p53-dependent $G_1$ arrest involves pRB-related proteins and is disrupted by the human papillomavirus 16 E7 oncoprotein. *Proc Natl Acad Sci USA* **91**: 5320–5324

Spiro IJ, Rice GC, Durand RE, Stickler R and Ling, CC (1984) Cell killing, radiosensitization and cell cycle redistribution induced by chronic hypoxia. *Int J Radiat Oncol Biol Phys* **10**: 1275–1280

Stokke T, Erikstein B, Smedshammer L, Boye E and Steen HB (1993) The retinoblastoma gene product is bound in the nucleus in early $G_1$ phase. *Exp Cell Res* **204**: 147–155

Teicher BA (1994) Hypoxia and drug resistance. *Cancer Metastasis Rev* **13**: 139–168

Tommasino M and Crawford L (1995) Human papillomavirus E6 and E7: proteins which deregulate the cell cycle. *BioEssays* **17**: 509–518

Weinberg, RA (1995) The retinoblastoma protein and cell cycle control. *Cell* **81**: 323–330

White RJ, Trouche D, Martin K, Jackson SP and Kouzarides T (1996) Repression of RNA polymerase III transcription by the retinoblastoma protein. *Nature* **382**: 88–90

Zariwala M, Liu E and Xiong Y (1996) Mutational analysis of the p16 family cyclin-dependent kinase inhibitors $p15^{INK4b}$ and $p18^{INK4c}$ in tumor-derived cell lines and primary tumors. *Oncogene* **12**: 451–455

*© Cancer Research Campaign 1998*